# Nerve-sparing versus non-nerve-sparing radical hysterectomy: surgical and long-term oncological outcomes

**DOI:** 10.18632/oncotarget.27078

**Published:** 2019-07-16

**Authors:** Antonio Gil-Moreno, Melchor Carbonell-Socias, Sabina Salicrú, Melissa Bradbury, Ángel García, Ramona Vergés, Oriol Puig Puig, José Luís Sánchez-Iglesias, Silvia Cabrera-Díaz, Javier de la Torre, Natalia R. Gómez-Hidalgo, Assumpció Pérez-Benavente, Berta Díaz-Feijoo

**Affiliations:** ^1^Unit of Gynecologic Oncology, Department of Obstetrics and Gynecology, Hospital Universitari Vall d’Hebron, Vall d’Hebron Barcelona Hospital Campus, Universitat Autònoma de Barcelona, Barcelona, Spain; ^2^Department of Pathology, Hospital Universitari Vall d’Hebron, Vall d’Hebron Barcelona Hospital Campus, Universitat Autònoma de Barcelona, Barcelona, Spain; ^3^Radiotherapy Department, Hospital Universitari Vall d’Hebron, Vall d’Hebron Barcelona Hospital Campus, Universitat Autònoma de Barcelona, Barcelona, Spain

**Keywords:** cervical cancer, morbidity, recurrence, surgical treatment, survival

## Abstract

**Objectives:**

There are controversies regarding the long-term oncological safety of preservation of pelvic innervation during radical hysterectomy (RH). This study aimed to analyze the feasibility and safety of nerve-sparing radical hysterectomy (NSRH) for cervical cancer compared with non-NSRH following 17 years of experience in a tertiary cancer referral center.

**Materials and Methods:**

Between May 1999 and June 2016, all patients who underwent RH for cervical cancer were followed-up prospectively. Comparison analyses regarding surgical outcomes, complications, overall survival (OS), disease-free survival (DFS), and cancer-specific survival (CSS) were performed between patients treated with NSRH and non-NSRH.

**Results:**

A total of 188 patients were included (113 non-NSRH and 75 NSRH). The median follow-up was 112 months. Estimated blood loss and hospital stay were all significantly lower in the NSRH group. Overall intraoperative complication rate (*p* = 0.02) and need for transfusion (*p* = 0.016) were lower in the NSRH group. There were no differences in the median operation time, OS, DFS, CSS, or recurrence rates between the NSRH and non-NSRH group.

**Conclusions:**

Our study provides a wide perspective on the developments of nerve-sparing procedures for the management of women with early-stage cervical cancer. Our results suggest that NSRH is a feasible and safe procedure, with reduced morbidity outcomes.

## INTRODUCTION

Open conventional abdominal radical hysterectomy without nerve-sparing has been for many years the primary treatment for patients with early stage cervical cancer [1, 2]. However, up to 25% of these patients will suffer severe reduction in their quality of life secondary to bladder, rectal, and sexual dysfunctions following the procedure [3]. This is mainly caused by the iatrogenic damage of the pelvic nerve plexus during surgery [4, 5].

With the aim to provide an alternative surgical technique for these patients, the preservation of pelvic innervation during radical hysterectomy (RH) was developed in Japan in the early 1920s [6]. This technique evolved in the 1960s by adding the division of the paracervix into its vascular and neural components, as well as sparing the nerve bundle when sectioning the uterosacral ligament [7]. This technique, called nerve-sparing RH (NSRH), was later expanded, allowing for the preservation of the splanchnic nerves (parasympathetic innervation), inferior hypogastric nerves (sympathetic innervation), and vesical branch of the lower hypogastric plexus during the dissection of the paracervical tissue. Initially, the use of NSRH was limited because of the difficulties in the identification of the anatomical structures and the lack of data regarding its oncological safety. In fact, it was not until 2008 that NSRH was first introduced in a standardized RH classification [8]. Progressively, it was adopted by some European centers [9–15] and over the last decade, it is has become more commonly performed in the USA by gynecologic oncologists following the widespread use of robotic technology.

However, as reflected by a recent systematic review, some groups still hesitate to introduce minimally invasive surgery (MIS) for NSRH, arguing that the heterogeneity in surgical techniques and the quality and volume of published literature prevents a high degree of certainty regarding both oncologic safety and quality of life improvement, compared with conventional RH [16].

We present the results from our institution of a study with one of the longest follow-up duration to date and analyze how the introduction of a nerve-sparing technique during RH has influenced the surgical and oncologic outcomes of these patients. This study aimed to analyze the feasibility and safety results of NSRH for cervical cancer compared with conventional RH following 17 years of experience in a tertiary cancer referral center.

## RESULTS

Between May 1999 and October 2006, a total of 188 patients diagnosed with early-stage cervical cancer underwent RH in our institution. Of these patients, 113 underwent a non-NSRH, 43 (38%) a Piver type II, and 70 (62%) a Piver type III RH. A nerve-sparing technique was carried out in all radical hysterectomies performed after October 2006 (*n* = 75): 30 (40%) with type B1 and 45 (60%) with type C1. The decision to perform RH in the five cases of locally advanced disease (IIB = 2 and IIB2-IIA2 = 3) was influenced by the patient’s characteristics and preferences and agreed by the gynecology-oncology team.

The median age was 48.2 (range, 25–77) years with a median body mass index of 26.1 (range, 18–40) kg/m^2^. The median follow-up was 112 (range, 53.7–162.3) months. The predominant histologic type was squamous cell carcinoma (61.17%), followed by adenocarcinoma (32.4%). Final pathology revealed 16 (8.51%) patients with tumors larger than 4 cm (pT1b2-T2a2) and 15 (7.98%) with microscopic parametrial involvement (pT2b) with no significant differences between the NSRH and non-NSRH groups. Characteristics of the study population and intra-operative data are presented in Tables 1 and 2.

**Table 1:** Clinical and pathologic data in the NSRH and non-NSRH groups

	NSRH *n* = 75	Non-NSRH *n* = 113	All patients *n* = 188	*p*
**Age, years mean (SD)**	47 (11.6)	49 (12.8)	48.21 (12.35)	0.27
**BMI, median (range)**	25 (18–37)	26 (18–40)	26.16 (18–40)	0.21
**MS, *n* (%)**	27 (36.49)	42 (37.7)	69 (36.9)	0.92
**Previous abdominal surgery, *n* (%)**	30 (52.2)	70 (59.2)	100 (53.1)	0.14
**Parity, median (range)**	2 (0–6)	2 (0–7)	2 (0–7)	0.52
**Histological type, *n* (%)**				
**Squamous cell carcinoma**	44 (58.6)	72 (62.8)	115 (61.2)	0.55
**Adenocarcinoma**	27 (36)	34 (30.1)	61 (32.4)	
**Others**	4 (5.3)	8 (7.06)	12 (6.38)	
**Histological grade, *n* (%)**				
**G1**	10 (13.3)	10 (8.85)	20 (10.6)	0.012
**G2**	44 (58.7)	56 (49.5)	100 (53.2)	
**G3**	21 (28)	33 (29.2)	54 (28.7)	
**NS**	0	14 (12.4)	14 (7.4)	
**FIGO stage, *n* (%)**				
**1A2**	3 (4)	8 (7.08)	11 (5.85)	0.27
**1B1 ≤ 2 cm**	27 (36)	34 (30.1)	61 (32.45)	
**1B1 > 2 cm**	34 (45.3)	63 (55.7)	97 (51.6)	
**IIA1**	6 (8)	8 (7.1)	14 (7.45)	
**IIB**	2 (2.67)	0	2 (1.06)	
**IB2 & IIA2**	3 (4)	0	3 (1.6)	

BMI, body mass index (kg/m^2^); MS, menopausal status; Non-NSRH, non-nerve-sparing radical hysterectomy; NSRH, nerve-sparing radical hysterectomy; NS, not specified; *p*, *p* value; SD, standard deviation.

**Table 2:** Operative data

Surgery, *n* (%)	NSRH *n* = 75	Non-NSRH *n* = 113	All patients *n* = 188	*p*
**Type of RH, *n* (%)**				NA
*B1 RH*	30 (40)			
*C1 RH*	45 (60)			
*Type II RH*		43 (38)		
*Type III RH*		70 (61)		
**Ovarian preservation, *n* (%)**	28 (37.3)	43 (38.05)	71 (37.1)	0.88
**Sentinel node procedure, *n* (%)**	53 (71)	68 (60.1)	121 (64.4)	0.87
**Approach, *n* (%)**				
*LRH*	39 (52)	51 (45.1)	90 (47.9)	
*ORH*	14 (18.6)	62 (54.8)	76 (40.4)	
*RRH*	22 (29.3)	0	22 (11.7)	
*LRH+RRH (MIS)*	61 (81.3)	51 (45.1)	112 (59.7)	
**Extracted pelvic nodes, median (range)**	20 (8–49)	20 (5–52)	20 (5–52)	0.95
*ORH*	21.5 (9–49)	20.5 (5–-52)		0.80
*MIS*	20 (8–37)	18 (8–51)		0.25
**Patients with positive pelvic nodes, *n* (%)**	13 (17.33%)	11 (9.75%)	23 (12.77%)	0.126
**Mean total parametrial volume, cm**^**3**^ **(SD)**	18.91 (9.62)	20.26 (10.47)	19.68 (10.1)	0.41
**Mean operative time, min. (SD)**	261 (47.98)	269 (50.7)	266.08 (49.68)	0.27
*ORH*	239.64 (53.65)	246.05 (38.78)		0.74
*MIS*	266.19 (45.56)	297.55 (49.37)		0.002
**Mean blood loss, cm**^**3**^ **(SD)**	199.32 (191.81)	460.93 (276.29)	357.41 (277.23)	< 0.0001
*ORH*	317.86 (302.94)	544.35 (309)		0.0006
*MIS*	171.67 (145.56)	359.51 (188.09)		0.0000
**Hospital stay (HS), median (range)**	3 (2–12)	8 (2–33)	5 (3–10)	< 0.0001
*ORH*	6 (3–12)	9 (5–33)		0.0000
*MIS*	3 (2–7)	5 (2–32)		0.0000

LRH, laparoscopic radical hysterectomy; NA, not applicable; NSRH, nerve-sparing radical hysterectomy; Non-NSRH, non-nerve-sparing radical hysterectomy; ORH, open radical hysterectomy; RRH, radical robotic hysterectomy; SD, standard deviation.

Estimated blood loss (EBL) and hospital stay (HS) were all significantly inferior in the nerve-sparing group, whereas no differences were found in operative time (OT), even when taking into account the surgical approach (open or MIS).

Overall intra-operative complication rate (*p* = 0.02) and the need for transfusion (*p* = 0.02) were significantly lower in the NSRH group. The conversion rate to laparotomy in the NSRH group was 0%, whereas for the non-NSRH it was 3.92%. Morbidity data are detailed in Table 3.

**Table 3:** Morbidity data

	Patients	Intraoperative complications *n* = 34	≤ 30 Days complications^a^ *n* = 37
**Nerve-sparing**		2 vesical lesions (*suture and prolonged urinary catheter)*	**Grade II: *n* = 4** 2 urinary infections 1 acute urine retention 1 abdominal wall infection
		1 ureteral section *(suture)* 3 blood transfusions	**Grade IIIa: *n* = 1** lymphocele (*drainage*)
	75		**Grade IIIb: *n* = 5** 2 vaginal dehiscences *(vaginal suture without complications)* 1 urinoma secondary to ureteral lesion (secondary to sutured ureter) (*laparotomy ureteroneocystostom)* 2 abdominal eviscerations
**Non-nerve sparing**		1 vesical perforation 2 ureteral sections (*ureteroneocystostomy)*	**Grade II *n* = 21** 7 urinary infections 6 acute urine retention 5 ileus 3 abdominal wall infections
		2 conversions to laparotomy *(due to ureteral section)* 1 anaphylactic shock secondary to Isosulfan Blue injection for SLN identification	**Grade IIIb: *n* = 5** 4 vaginal dehiscence (vaginal suture)1 urinary fistula
	113	1 intestinal perforation 1 obturator vein lesion (*bipolar coagulation and compression)* 1 cava vein lesion *(suture)* 1 left internal Iliac vein lesion *(Tissucol*™ *-Baxter AG, Vienna, Austria- and compression)* 1 external iliac vein lesion *(suture)* 17 blood transfusions	**Grade V: *n* = 1** Progressive multiorgan dysfunction secondary to massive intraoperative bleeding due to vascular lesion

Treatments within parentheses in italics. SLN, sentinel lymph node.

^a^Clavien-Dindo Scoring System [20].

The bladder and bowel function were investigated at one year after surgery. A total of 7 patients in the NSRH group and 22 patients in the non-NSRH presented bladder and rectal functional-related complications. The results are shown in Table 4. The incidence of urinary incontinence and loss of bladder filling sensation was significantly lower in the NSRH group compare to the non-NSRH group (1.3% vs. 3.5%; 1.3% vs. 5.3%, respectively) (*p* > 0.05).

**Table 4:** Bladder and rectal function 12 months after surgery

	NSRH (*n* = 75)	Non-NSRH (*n* = 113)	*p*-value
**Urinary incontinence**	1 (1.3%)	4 (3.5%)	0.81
**Loss of bladder filling sensation**	1 (1.3%)	6 (5.3%)	0.48
**Straining of urinate**	3 (4%)	8 (7%)	0.76
**constipation**	2 (2.6%)	4 (3.5%)	0.55
**Total**	7 (9.3%)	22 (19.4%)	0.06

**Figure 1:**
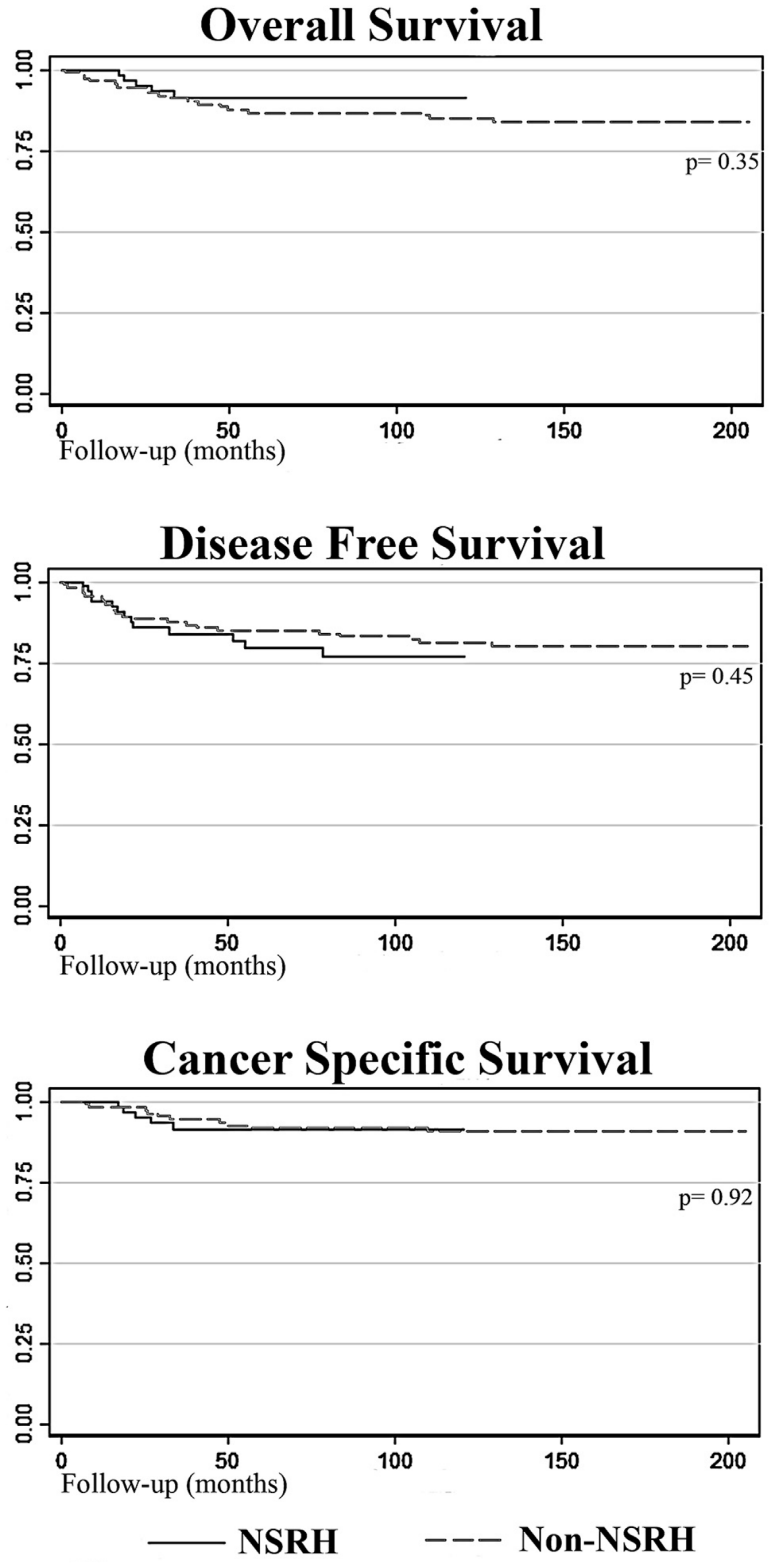
Survival curves comparing NSRH with non-NSRH.

In terms of oncologic outcomes, there were no differences in OS, disease-free survival (DFS), or recurrence rates between the NSRH and non-NSRH groups (Figure 1). Of the 188 patients, 156 (82.98%) where alive and disease-free at the end of the study period, that is, 65 (86.67%) in the NSRH group and 91 (80.53%) in the non-NSRH group (*p* = 0.18). Recurrences are specified in Table 5. Cancer-specific mortality rate was 6% for the NSRH and 8.8% for the non-NSRH (*p* = 0.91).

**Table 5:** Recurrence data

	NSRH *n* = 75	Non-NSRH *n* = 113	Total *n* = 188
**Recurrence location, *n* (%)**			
Vaginal	3 (23.08%)	3 (21.43%)	6 (21.4%)
Pelvic wall	5 (38.46%)	4 (21.43%)	9 (32.1%)
Peritoneal	1 (7.69%)	1 (7.14%)	2 (7.1%)
Supraclavicular lymph node	1 (7.69%)	0	1 (3.5%)
Distant metastasis	2 (15.38%)	1 (7.14%)	3 (10.7%)
Pelvic wall + vaginal	0	3 (21.43%)	3 (10.7%)
Pelvic wall + umbilical	0	1 (7.14%)	1 (3.5%)
Peritoneal + inguinal lymph node	0	1 (7.14%)	1 (3.5%)
Hepatic + vaginal	0	1 (7.14%)	1 (3.5%)
Pelvic wall + distant metastasis	1 (7.69%)	0	1 (3.5%)
**Time of recurrence, *n* (%)**			
< 1 year	4 (30.8%)	6 (40%)	10 (35.7%)
< 2 years	7 (53.8%)	12 (80%)	19 (67.9%)
< 5 years	10 (76.9%)	14 (93.3%)	24 (85.7%)
Total, *n* (100%)	13 (100%)	15 (100%)	28 (100%)

## DISCUSSION

Evidence following publication of different series [17, 18] and recent meta-analysis [19, 20] has shown that NSRH decreases bladder and rectal dysfunction without compromising recurrence or survival rates compared with non-NSRH. This study represents one of the longer-term follow-up periods published to date in which the results for non-NSRH and NSRH are compared with respect to surgical and oncologic outcomes. Although over 80% of nerve-sparing procedures were performed by MIS, there were no differences in terms of surgical time when compared with the non-NSRH group. Moreover, within the MIS group, NSRH presented with a significant reduction in OT. These results differ from those presented in a recent meta-analysis [19, 21] in which both laparoscopic and abdominal routes presented greater surgical times in the nerve-sparing group. This difference could be partially explained by the introduction of the RRH in our series, which presented with similar surgical times to ORH and significantly lower times than LRH, contributing to an overall reduced OT in MIS.

Similarly, in our series, NSRH showed significantly inferior EBL compared to that of non-NSRH group. It could be argued that the significant difference in EBL could be explained by the fact that most NSRH were performed by MIS. The reduction in EBL with this approach is consistently reported in most studies [22]. However, the reduction in EBL is persistent even when comparing NSRH with non-NSRH within each of the surgical approaches (MIS and ORH). In this regard, the accumulative experience of the surgical team as well as the progressive introduction of vessel sealer devices could partially explain the results [23].

The number of excised pelvic nodes in our series is similar to that previously reported in the literature, with no differences between the NSRH and non-NSRH groups. In two published meta-analysis [19, 24] in which NSRH is compared to non-NSRH, the number of excised pelvic nodes is not considered a meaningful variable of comparison. Regardless, we consider that these results would support the oncologic safety of the nerve-sparing technique.

In our series, the total paracervix volume resected was similar between the NSRH and non-NSRH groups, regardless of the surgical approach. These results are similar to those presented by other groups [24, 25]. Thus, nerve-sparing techniques do not appear to be associated with smaller surgical resections in laparoscopic nor open procedures.

In our series, we found significant differences in vascular injury rates and patients requiring transfusion, in favor of the NSRH group. Although our study supports the evidence presented in a recent meta-analysis [19] regarding the reduction in intraoperative complications associated with nerve-sparing techniques, we should reconsider the fact that most of the nerve-sparing procedures were performed by MIS, which by itself is related to fewer intra-operative complications [25]. We decided to consider transfusion as a complication because it marked a differential management of the patients and could be related to greater postoperative morbidity.

The NSRH technique improved all the urinary symptoms (frequent urination, urinary incontinence, difficulty emptying and bladder sensation), which could be due to the complete preservation of autonomic nervous structure that controls the bladder in the nerve plane [26]. Moreover, the full recovery of urinary function usually take approximately one year after radical surgery; according to recent publications [27] we found that the NSRH group presented less urinary incontinence, loss of bladder filling sensation and straining of urinate compare to non- NSRH group although it did not reach statistical significance.

Our results show an association between NSRH and significant HS reduction. Morbidity reduction related to autonomic nerve plexus preservation as well as progressive improvement of the surgical technique with accumulated experience may account for these results. Nevertheless, we consider that the changes in the peri-operative management of these patients, with the progressive introduction of enhanced recovery after surgery (ERAS^®^) in our Gynecology Oncology unit in recent years, probably played a fundamental role in these results [28]. Our oncologic results in terms of OS, DFS, recurrences and cancer-specific mortality rates were similar between the two groups, as suggested by previous studies [24].

We know that there are some limitations in our study. First, it was conducted in a single centre and, therefore, it could be subject to biases derived from the technology and expertise available in our centre. Second, we had few patients to generate conclusive results in some aspects of the study. And third, as the study was carried out for more than 17 years there were unavoidable changes in the methodologies used. In contrast, one of the strengths of this work is that it reflects our long-term experience in these surgical techniques and the long follow-up of a large number of patients.

## MATERIALS AND METHODS

### Patients

The study was approved by our institutional review board (PR (AMI) 23/1999) and in accordance with the Helsinki Declaration. All women provided informed consent for the surgical procedure, as well as for the recording and analysis of all relevant clinical data.

Between May 1999 and June 2016, all patients who were diagnosed with International Federation of Gynecology and Obstetrics (FIGO) stage IA2-IB1-IIA1 cervical cancer who underwent an RH as first treatment at our institution were included in the study. Pregnant women and those patients who had received previous chemotherapy or pelvic radiotherapy were excluded from the study.

All patients had their initial pathologic diagnosis confirmed at our institution, following a cervical punch biopsy or a cone biopsy. Patients were staged according to the 2009 cervical cancer FIGO classification. Patients undergoing surgery before 2009, which had been initially staged according to the 1988 FIGO classification, were restaged according to the new 2009 staging system [1, 2]. In all patients, magnetic resonance imaging was performed prior to surgery. The surgical complications were classified according to Dindo et al. [29].

Information of interest was recorded prospectively from the time of surgical indication to the last follow-up date recorded at the time of study closure.

### Treatment

Nerve-sparing technique was first introduced in our institution in October 2006. Procedures performed before 2006 were classified following the 1974 RH classification [30], and after the introduction of nerve-sparing technique, the new 2008 classification [15] was used accordingly. Patients with FIGO stage IA2 or IB1 with tumor size ≤ 2 cm underwent proximal or modified RH (Piver type II) or type B1. Patients with FIGO stage IB1 with a tumor mass less than 2 cm on physical examination but with a larger mass on magnetic resonance imaging and those with a tumor mass larger than 2 cm underwent distal RH technique (Piver type III) or type C1.

The decision to perform a laparoscopic or open approach depended on the patient’s characteristics together with the surgeons’ and patients’ preferences. In 2009, with our center’s acquisition of one of the first robotic surgery devices in our country, robotic radical hysterectomy (RRH) was progressively introduced for performing RH. General exclusion criteria for the MIS approach (laparoscopic or robotic) include severe cardiorespiratory disease preventing a Trendelenburg position, enlarged uterus over 12 gestational weeks, body mass index of 40 kg/m^2^ or higher, and age 80 years or older. The decision to use robotic surgery was also limited by the availability of the device.

### Surgical technique

In the nerve-sparing technique (radical hysterectomy C1), the inferior hypogastric nerves, pelvic splanchnic nerves, and inferior hypogastric plexuses with efferent nerves to the bladder and vagina are preserved, as opposed to the conventional technique. The key to optimal nerve preservation is the systematic identification of the anatomical structures of the autonomic nervous system. The minimally invasive approach can facilitate this surgical procedure in comparison to the laparotomic approach.

**Figure 2:**
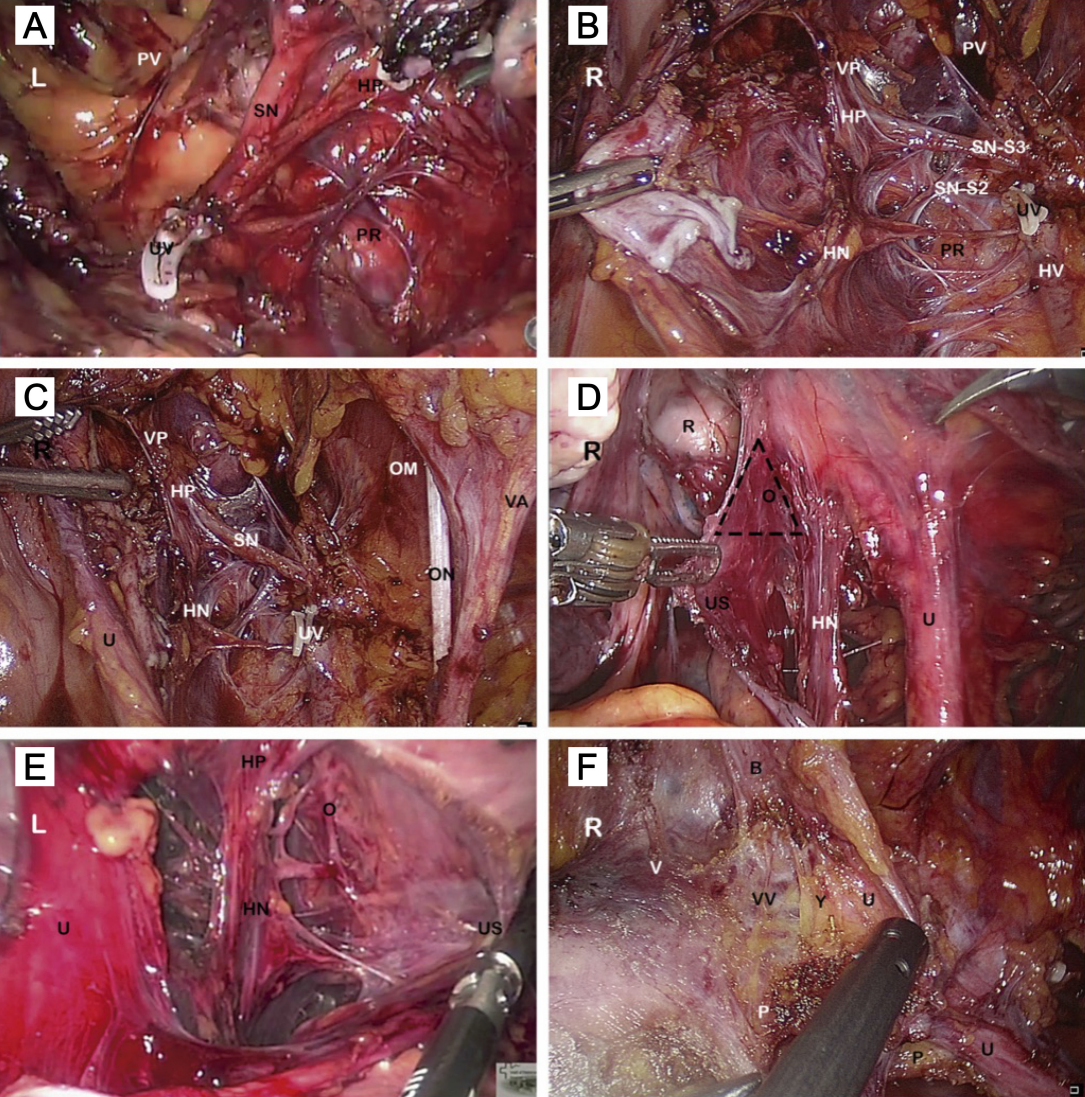
Nerve-sparing radical hysterectomy: Key steps. R right side view of the pelvis; L left side view of the pelvis; B bladder; HN inferior hypogastric nerve; HP inferior hypogastric plexus; HV hypogastric vein; O Okabayashi space; OM intern obturator muscle; ON obturator nerve; P paracervical tissue; PR pararectal space; PV paravesical space; R rectum; SN pelvic splanchnic nerve (S2-S3); U ureter; US uterosacral ligament; UV deep uterine vein stump (hemolock); V vagina; VA superior vesical artery; VP vesical plexus; VV inferior and middle vesical veins; Y Yabuki space. (**A**) The pelvic splanchnic nerves are seen joining the HP in a perpendicular fashion, after sectioning the UV. (**B**) Distal fibers of the HP run toward the vagina and bladder along the dorsal vesicouterine ligament and the lateral wall of the vagina. (**C**) Pelvic autonomic nerves, nerve-sparing radical hysterectomy. (**D**) Isolation and preservation of the HN during dissection of the US and the Okabayashi space. (**E**) Dissection of the US and the Okabayashi space. (**F**) Dissection of the ureteral tunnel and Yabuki space.

Three fundamental steps should be kept in mind when developing the NSRH technique (Figure 2). The first step is to isolate and separate the deep uterine vein from the lower hypogastric plexus. After sentinel lymph node (SLN) identification and subsequent pelvic lymphadenectomy, the paravesical and pararectal spaces are dissected. This isolates the paracervical tissue that includes the uterine artery and the superficial and deep uterine veins. When dissecting, coagulating, and sectioning the deep uterine vein, ventral branches of the splanchnic nerves (S_2_-S_3_-S_4_) can be observed along its path toward the lower hypogastric plexus (Figure 2A–2C). The second step includes the isolation and preservation of the inferior hypogastric nerves, which are attached to the splanchnic nerves in the inferior hypogastric plexus, during dissection of the uterosacral ligament and the Okabayashi space (Figure 2D, 2E). Finally, the third step involves the preservation of the efferent bladder branches of the inferior hypogastric plexus, on the posterior leaf of the vesico-uterine ligament, during dissection of the Yabuki space. Visualization of the middle and lower vesical veins helps preserve these efferent branches (Figure 2F).

The surgical technique of open radical hysterectomy (ORH), laparoscopic radical hysterectomy (LRH), and RRH with and without a nerve-sparing approach together with the SLN identification procedure used by our group has been described in detail in previous publications [14, 31, 32].

### Statistical analysis

Data are expressed as mean and standard deviation for normally distributed variables and as median and range of variables whose distribution departed from normality. Variables were tested for normality using the Kolmogorov-Smirnoff test. For qualitative variables, chi-square and Fisher’s exact test were used. For quantitative variables, analysis of variance or Kruskal-Wallis test was used. Kaplan-Meier survival analyses were performed to estimate overall survival (OS), recurrence-free survival, and cancer-specific survival. Differences in the probability of survival according to the surgical procedure were compared using the log-rank test. Statistical significance was set at *p* < 0.05. STATA^®^ 13.1 (StataCorp; College Station, TX, USA) was used for the statistical analysis.

## CONCLUSIONS

In conclusion, our study showed that NSRH in early-stage cervical cancer is a feasible alternative. Our results indicate that it is associated with similar OS, DFS, recurrence rates, and cancer-specific mortality rates compared with non-NSRH.

This paper defines what has been the evolution of radical hysterectomy in the management of early-stage cervical cancer for the last two decades in our institution. We believe that the lack of data regarding OS and DFS after long periods of follow-up that conditioned the expansion of NSRH surgery has been properly addressed by the long follow-up period in this study. Properly designed prospective clinical trials about the subject would offer stronger evidence to support NSRH as the preferred approach for early-stage cervical cancer.

## Abbreviations

CSS: Cancer-specific survival
DFS: Disease-free survival
EBL: Estimated blood loss
FIGO: Federation of Gynecology and Obstetrics
HS: Hospital stay
LRH: Laparoscopic radical hysterectomy
MIS: Minimally invasive surgery
NSRH: Nerve-sparing radical hysterectomy
ORH: Open radical hysterectomy
OS: Overall survival
OT: Operative time
RH: Radical hysterectomy
RRH: Robotic radical hysterectomy
SLN: Sentinel lymph node
